# Supplementary material for: Complete structure of the chemosensory array core signalling unit in an E. coli minicell strain
Source: Nat Commun. 2020 Feb 6;11:743. doi: 10.1038/s41467-020-14350-9 (PMC7005262; doi:10.1038/s41467-020-14350-9)
Supplement: Supplementary file 1 — Supplementary information [file 41467_2020_14350_MOESM1_ESM.pdf]

SUPPLEMENTARY INFORMATION

Complete structure of the chemosensory array core signalling unit in an *E. coli* minicell strain

Burt[#], Cassidy[#] et al.

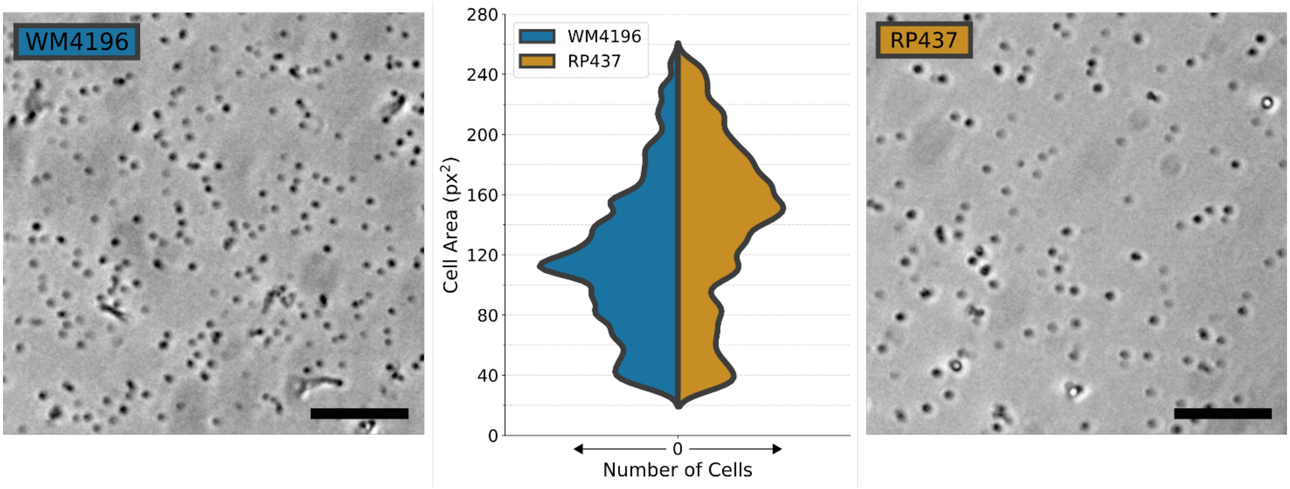

Supplementary Figure 1: Size comparison of WM4196 and UU3118 minicells.

Minicells from WM4196 and UU3118 cultures were purified by differential centrifugation as described in Methods for the FRET kinase assay, immobilized on a polylysine-coated coverslip, and imaged at the same magnification by phase contrast microscopy (left and right panels, scale bar = 8 µm). After passing the images through a threshold filter to facilitate cell measurements, ImageJ software and its plug-in MicrobeJ[1] were used to identify hundreds of minicells, define their edges and calculate their 2-D surface areas from their dimensions. To minimize the background of non-minicells or inaccurate measurements, we implemented arbitrary cutoffs of areas below 0.15 µm^2 and above 1.35 µm^2. Probability density functions for the minicell areas of each strain were estimated using kernel density estimation (n=613 for each strain) in seaborn (https://seaborn.pydata.org/generated/seaborn.kdeplot.html) with a kernel bandwidth 1/10th the size of the standard deviation of the data for each strain. The measured areas may be overestimated due to optical artifacts and resolution limits but should be comparable between strains.

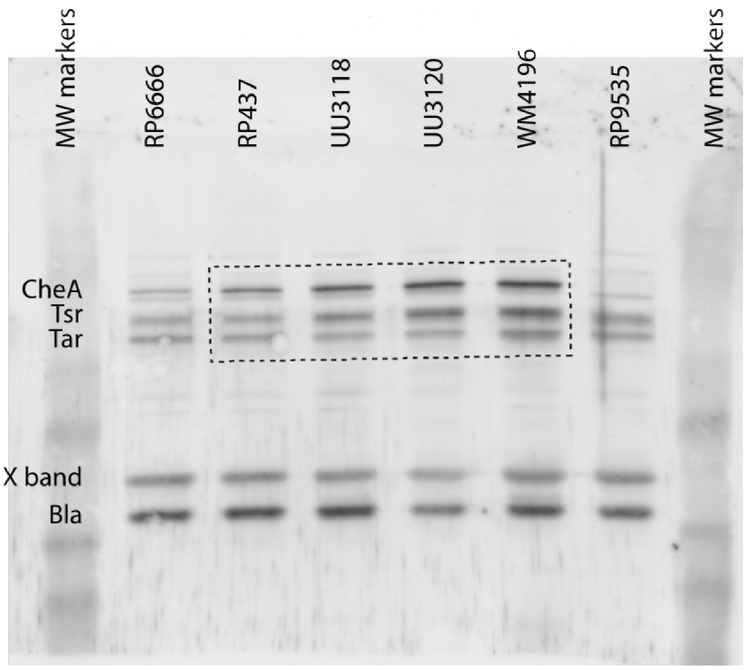

Supplementary Figure 2: Expression of three principal chemosensory array components: the Tsr and Tar chemoreceptors and the CheA kinase.

Tar and Tsr are identified based on their molecular weights and gel mobility behaviors. CheA bands are identified by their absence in strain RP9535, which has a deletion of the *cheA* gene but has the *tsr* and *tar* genes. The Bla and X bands were used as loading controls. Note that their relative intensities are constant across all sample lanes.

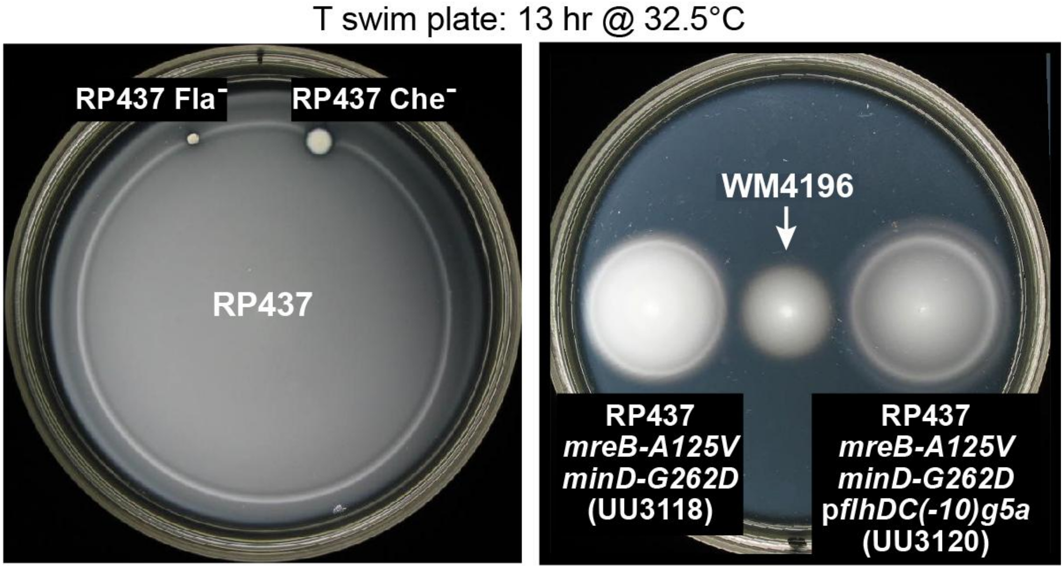

Supplementary Figure 3: Chemotaxis performance of RP437 and WM4196 strains.
Cells were streaked on T hard plates (T broth plus 14 g/L agar) and grown for 24 hours (WM4196) or 18 hours (RP437 strains) at 37°C, Colonies were picked to T swim plates (T broth plus 2.5 g/L agar) and incubated at 32.5°C for 13 hours. Chemotactic colonies exhibit one or more dense bands or rings of cells that track metabolism generated nutrient gradients in the plate.

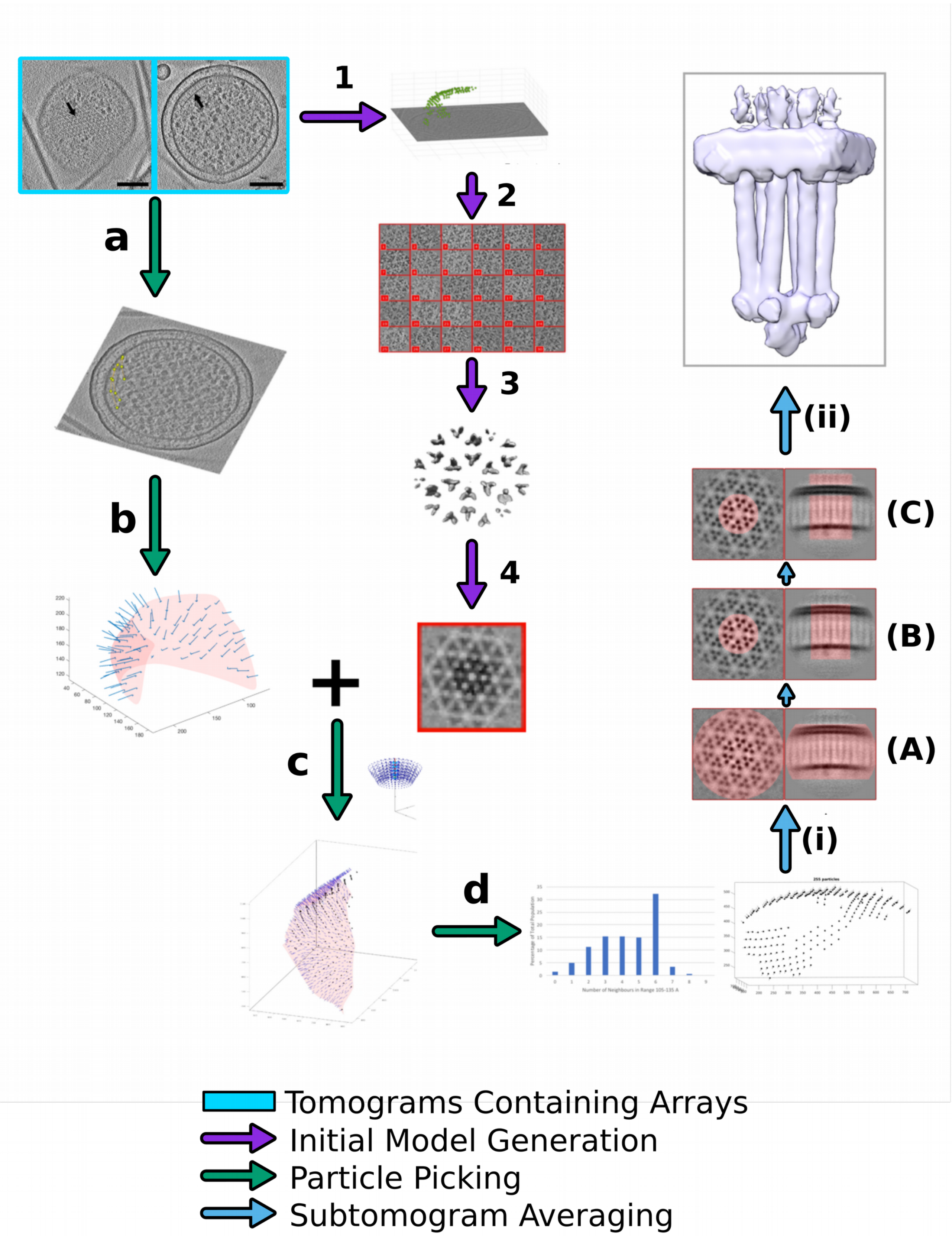

Supplementary Figure 4: Overview of Initial Model Generation, Particle Picking and Subtomogram Averaging Workflows.

The initial model generation workflow consists of manual picking of the centers of ToD-hexagons in the tomograms (1), extraction of picked particles (2) followed by manual alignment and averaging before subtomogram averaging to produce an initial reference (3). The average was filtered to 45 Å to serve as

an initial reference. The particle picking workflow starts with the definition of a surface model following the curvature of the inner membrane in areas where chemoreceptor arrays are visible (a). Points are generated on these surface models with initial orientation estimates given by the normal to the surface model, oversampled relative to the expected spacing of ca. 120 Å between hexagon centres (b). Particles are extracted at each point and aligned to our initial model from the initial model generation workflow, constraining the possible tilt angles to a 60 degree cone and the XY translations to 120 Å (c). After constrained alignment, only particles with more than three nearest neighbours at a distance of 120+-15 Å are retained (d). The remaining particles are then introduced into a subtomogram averaging workflow (i) comprising 16 iterations of global alignment inside a mask including higher-order array structure over 573 Å (A), 16 iterations of local alignment inside a mask including 3 CSUs and the membrane but no periplasmic density (B) and 16 iterations of local alignment inside a mask including 3 CSUs, without the membrane and with density in the periplasmic space (C). This is followed by local resolution estimation and subsequent local resolution filtering before centering the reconstruction on the C2 axis of one CSU and applying C2 symmetry (ii). See also Methods.

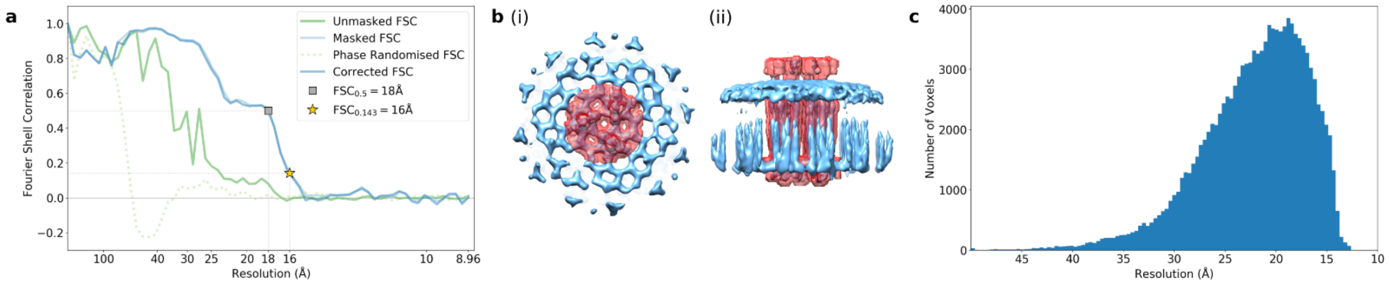

Supplementary Figure 5: The resolution of the cryo-ET map as determined by the Fourier Shell Correlation.

The FSC is calculated between two masked half-maps (light blue), unmasked half-maps (containing densities not included during alignment) (green) and an FSC between two masked half-maps for which the phases of the Fourier transform were randomised beyond the point in which the FSC dropped below 0.8 (light green - dashed). The latter curve is used to validate the masking and produces a corrected FSC (blue) which does not significantly deviate from the FSC calculated between two masked half maps. The extent of the mask used for masked FSC calculation is shown (b) for the XY plane (i) and the YZ plane (ii) of our reconstruction. The local resolution was estimated for the whole reconstructed area, from which we show a histogram of the local-resolution estimates inside the mask used for the masked-FSC calculations (c).

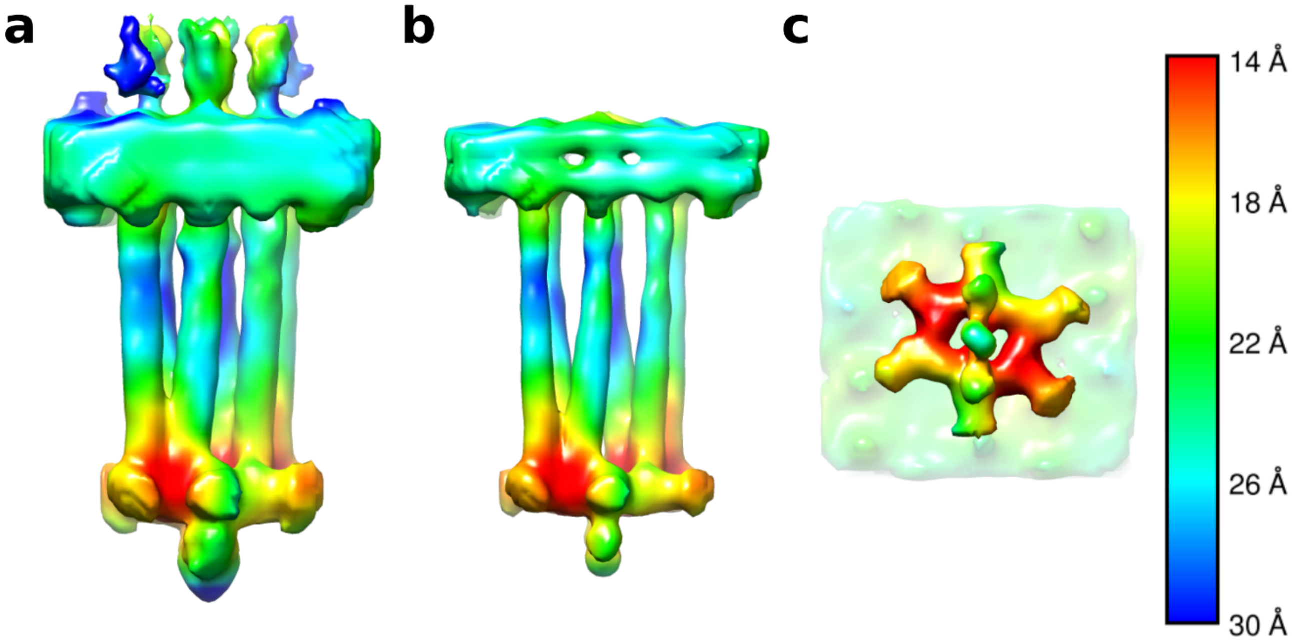

Supplementary Figure 6: Local resolution estimates of the cryo-ET map of the *E. coli* CSU.
Local resolution estimates are plotted onto isosurface renderings of the reconstruction. Isosurfaces are shown from the side at thresholds of 0.0045 (a), 0.018 (b) and from the bottom at a threshold of 0.018 (c), highlighting significant variation in local map quality.

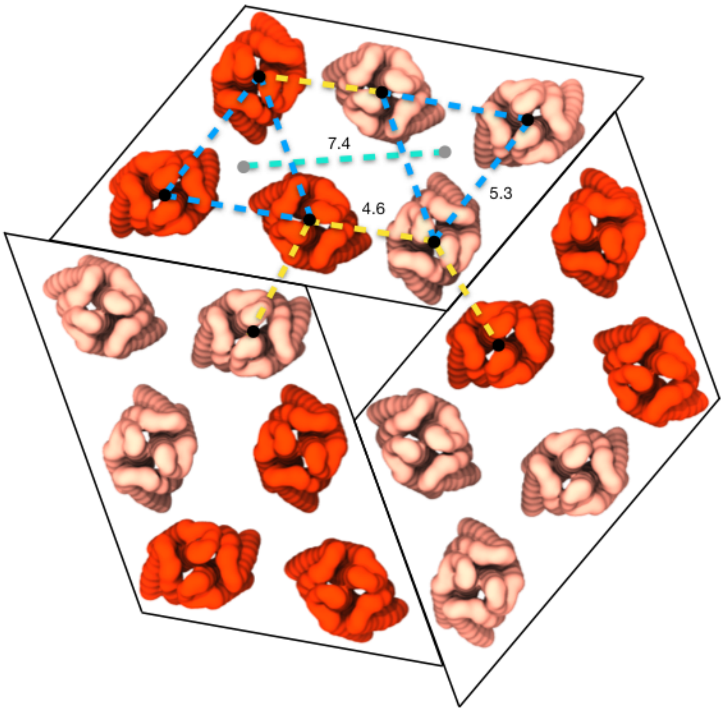

Supplementary Figure 7: Extended organization of receptor periplasmic domains

Distances between receptor periplasmic domains within and between core signalling units (CSUs) in the extended array. Individual core signalling units are boxed by a parallelogram with comprising receptor trimers of dimers (ToDs) colored in red and salmon. Distances are given in nanometers and are computed between the centers-of-mass of the receptor periplasmic domains (black circles) and the ToD symmetry axes (gray circles). Dashed lines of the same color denote equal distances. The model of the extended array architecture shown here was constructed by rigidly docking the MDFF-refined CSU model (Fig. 4) into the density map for the array centered on a hexagonal arrangement of 3 CSUs.

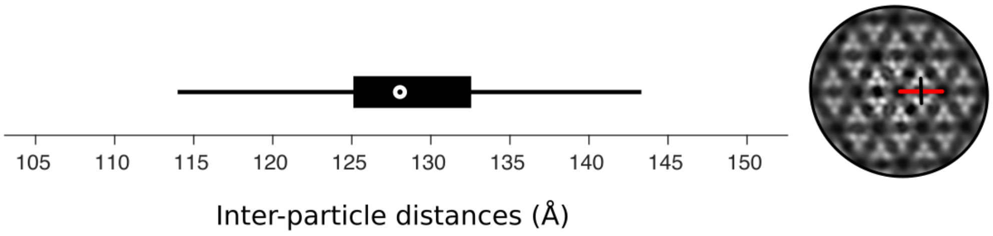

Supplementary Figure 8: Distribution of inter-particle distances in tomograms

A box plot to show the distribution of inter-particle distances in tomograms of WM4196 minicells. The central mark indicates the median (128 Å), the left and right edges of the box indicate the 25th and 75th percentile respectively. Whiskers extend to approximately 2.7σ. Particle positions (CheA/W ring centers) were found by subtomogram averaging. Inter-particle distances measured correspond to the red line shown on a 5 nm projection through the density of our reconstruction. The 7.4 nm inter-ToD distance corresponds to the black line perpendicular and above the red line.

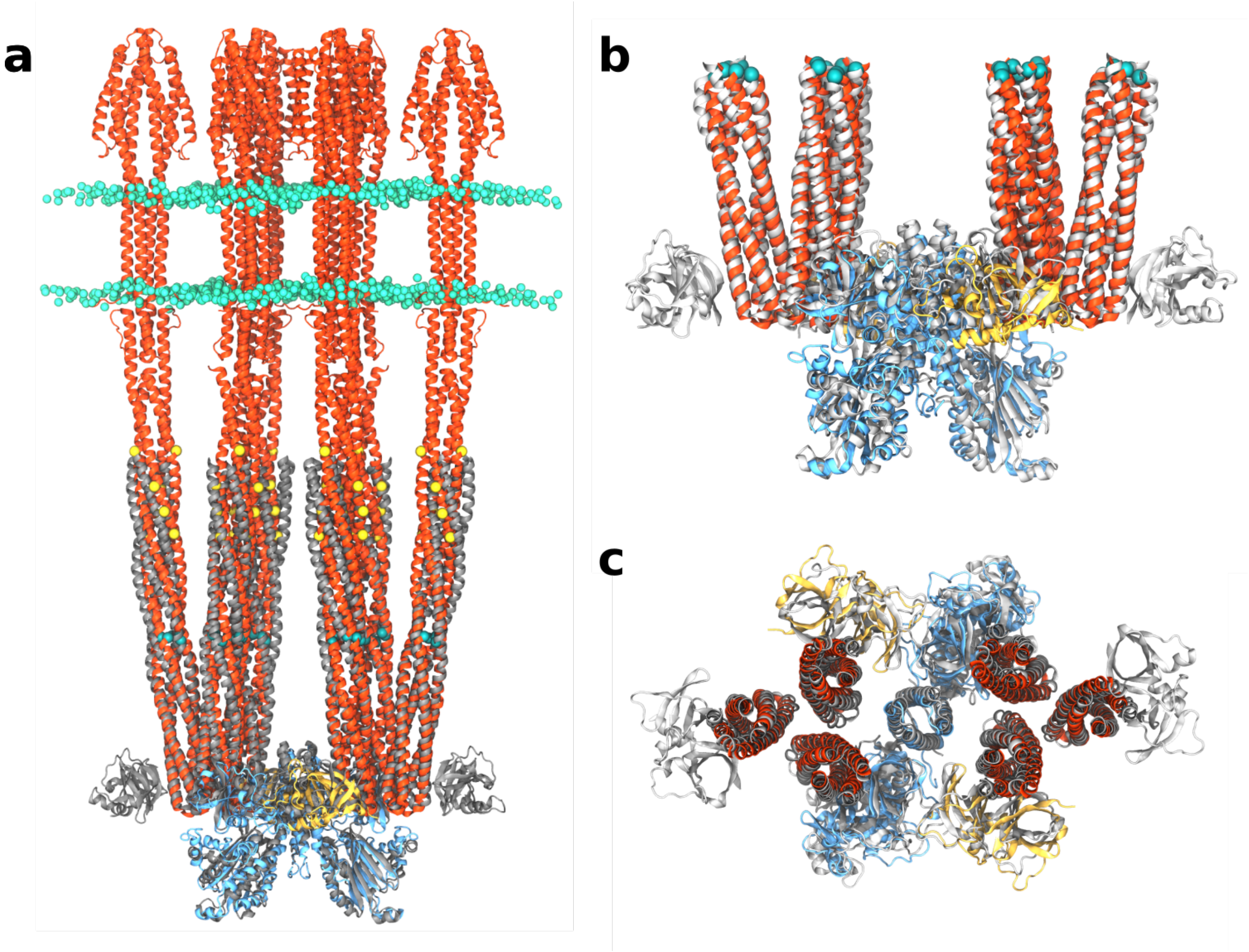

Supplementary Figure 9: Comparison between the *E. coli* transmembrane CSU model and a previously published model of the *T. maritima* CSU based on a cryo-ET map of *in vitro* reconstituted *E. coli* CSU (PDB 3JA6). (a-c) Overlay of the current *E. coli* CSU model with PDB 3JA6 (shown in grey) in full view from the side (a) as well as centered on the baseplate from the side (b) and top (c). The overlay was obtained by structural alignment of corresponding receptor regions in each model. Only the portion of the *T. maritima* receptors contained within the *in vitro* map and explicitly refined[2] is shown (see PDB 3JA6 header). Densities corresponding to two flanking CheW monomers in PDB 3JA6 were not included in the current *E. coli* CSU model. Colour scheme for the *E. coli* CSU model as in other figures.

Supplementary References

1. Ducret, A., Quardokus, E. M. & Brun, Y. V. MicrobeJ, a tool for high throughput bacterial cell detection and quantitative analysis. Nat. Microbiol. (2016). doi:10.1038/nmicrobiol.2016.77

2. Cassidy, C. K. et al. CryoEM and computer simulations reveal a novel kinase conformational switch in bacterial chemotaxis signaling. Elife 4, 1–20 (2015).
