# Supplementary material for: Complete structure of the chemosensory array core signalling unit in an E. coli minicell strain
Source: Nat Commun. 2020 Feb 6;11:743. doi: 10.1038/s41467-020-14350-9 (PMC7005262; doi:10.1038/s41467-020-14350-9)
Supplement: Supplementary file 5 — Description of Additional Supplementary Files [file 41467_2020_14350_MOESM5_ESM.pdf]

Title : Supplementary Movie 1: WM4196 tilt-series, tomogram and the derived map and model.

Description: A movie to show a tilt-series which contributed to the final reconstruction, the corresponding tomographic reconstruction and the in situ core-signalling unit density, the derived MDFF equilibrated model and the fit of the model in the density. The threshold for the surface representation of the model is lowered during the rotation to accentuate the presence of the periplasmic ligand-binding domains.
